# A Review of Adversarial Example Detection in IoT Sensor Networks: Methods, Evaluation, and Edge Deployment Constraints

**DOI:** 10.3390/s26165044

**Published:** 2026-08-08

**Authors:** Wenqiang Xu, Jian Li

**Affiliations:** School of Cyberspace Security, Beijing University of Post and Telecommunications, Beijing 100876, China; xuwenqiang@bupt.edu.cn

**Keywords:** IoT sensor networks, adversarial example detection, adaptive attacks, edge deployment, detection evaluation, formal certification

## Abstract

Deep learning has been widely deployed in critical scenarios such as the Internet of Things (IoT), industrial sensing, network intrusion detection, and cyber-physical system monitoring, where model inference directly affects system security, operational reliability, and service continuity. However, existing adversarial example detection studies remain insufficient for practical IoT deployment, as their validation often overlooks endpoint resource constraints, heterogeneous data modalities, physical environmental interference, communication protocol specifications, adaptive attacks, and adversary capability models. Moreover, detection outcomes are rarely connected with deployment locations, computational overhead, formal security assurance, and subsequent response strategies, which limits their engineering applicability. To address these limitations, this review systematically synthesizes recent representative studies in adversarial example detection and constructs a unified analytical framework integrating detection evidence, IoT deployment feasibility, and adaptive-attack evaluation. Based on the source of detection evidence, existing methods are categorized into input-consistency-based, feature-statistics-based, predictive-uncertainty-based, model-reconstruction-based, runtime-context-aware, and multi-strategy fusion detection, while formal certification is discussed as an independent security-assurance dimension. The review further analyzes the principles, applicable conditions, limitations, compatibility conflicts with IoT deployment constraints, and typical failure modes of these methods. The analysis identifies four key challenges: the lack of IoT-native adaptive evaluation, limited anomaly-boundary identification and cross-modal generalization, insufficient deployment-time security assurance, and weak coordination between detection decisions and security responses. Future research should therefore emphasize feasible attack paradigms, hierarchical lightweight detection, reliable multimodal fusion, certifiable operational boundaries, and auditable end-to-end response mechanisms, thereby supporting the evaluation and deployment of adversarial example detection in IoT scenarios.

## 1. Introduction

Machine learning models have gradually evolved from laboratory prototypes into key components of IoT sensing and decision-making systems. Contemporary IoT platforms, industrial control networks, smart cameras, radio-frequency signal recognition devices, acoustic monitoring systems, and network traffic analysis platforms transform heterogeneous information, including images, sounds, wireless signals, time-series measurements, and network traffic, into alarms, predictions, or control commands. As machine learning is increasingly involved in security-critical decisions, the system attack surface has expanded from identity authentication, data encryption, secure boot, and conventional intrusion detection to model inputs and inference processes. Attackers may not only exploit traditional network vulnerabilities to compromise devices but may also manipulate model inputs to alter inference outcomes, thereby disrupting monitoring, recognition, and control functions. Prior IoT studies have shown that device heterogeneity, hardware resource limitations, privacy protection, and distributed communication have long constrained secure system operation [1,2,3,4,5,6,7], while adversarial machine learning research has further demonstrated that even models with high classification performance may be misled by carefully designed small or structured perturbations [8,9,10,11,12,13,14,15,16,17,18,19,20].

Adversarial example detection is an important mechanism for improving model security and robustness. Unlike approaches that directly modify or retrain the classifier, detectors usually evaluate the suspiciousness of an input by analyzing raw inputs, latent feature representations, predictive probability distributions, reconstruction residuals, temporal relationships, or protocol behaviors, and then trigger responses such as input rejection, decision deferral, escalated review, or secondary verification. This modular characteristic is particularly suitable for IoT scenarios, because detection functions can be deployed on sensing terminals, edge gateways, cloud security centers, or collaborative monitoring layers without replacing already deployed task models. Nevertheless, high detection accuracy does not imply that a detector is necessarily trustworthy in operational systems. The evidence on which detection relies must be obtainable at the corresponding deployment layer, its computational and storage overhead must satisfy the resource budget, and its effectiveness must be validated when the attacker is aware of and able to adapt to the complete classifier-detector system.

On this basis, this review focuses on three interrelated problems in existing research. First, a clear gap remains between algorithm design and practical application scenarios. Many detection methods are validated on static image datasets and idealized digital perturbations, without sufficiently considering the resource limitations of IoT devices, the non-stationarity of sensing signals, and the realizability of perturbations in physical environments and communication protocols. Second, current evaluation and validation practices remain insufficient. The use of non-adaptive attacks, threshold tuning around specific attack types, or the reporting of isolated area under the receiver operating characteristic curve (AUROC) values may overestimate detector security in realistic adversarial environments; when an attacker can jointly optimize against the detection mechanism, the detection performance of some methods may decline substantially [18,21,22]. Third, detection methods are insufficiently connected with system operation mechanisms. Existing studies often report computational overhead, deployment location, trust assumptions for collaborative nodes, and detection performance separately, but rarely analyze how detection scores trigger subsequent security responses or how response processes affect system security, availability, and service continuity.

These problems are not independent; together they determine whether a detection method has practical application value. A lightweight detector is deployable only if it remains effective under feasible adaptive attacks. A method with strong statistical detection performance is still difficult to operate if its evidence cannot be obtained on the target device or if its computational cost exceeds the edge-node resource budget. Similarly, even when a detector accurately identifies suspicious inputs, inappropriate threshold selection or a response mechanism that frequently interrupts normal services may introduce new availability risks. Therefore, adversarial example detection should not be regarded merely as an isolated binary classification problem; it should be evaluated within a system framework jointly formed by attack conditions, deployment environments, resource constraints, and security responses.

Accordingly, the core argument of this review is that conclusions about adversarial example detection in IoT scenarios are sufficiently credible only when detection evidence, IoT deployment constraints, and attacker-aware evaluation protocols are specified simultaneously. Around this argument, this paper makes four contributions. First, existing methods are classified according to the evidence sources used by detectors, and formal certification is distinguished from empirical detection methods so that their differences and complementarity in security assurance can be clarified. Second, natural distribution drift, out-of-distribution inputs, and adversarial examples actively optimized by attackers are differentiated, and their differences in generation mechanisms, detection objectives, and security implications are analyzed. Third, attacker capabilities, perturbation feasibility, and knowledge assumptions are linked with evaluation metrics, detection thresholds, deployment costs, and security responses, thereby constructing a unified framework that covers both algorithmic performance and engineering feasibility. Fourth, four key research gaps are summarized, namely adaptive evaluation, anomaly boundaries and cross-modal generalization, deployment-stage security assurance, and detection-response coordination, and corresponding future directions are proposed.

**Figure 1:**
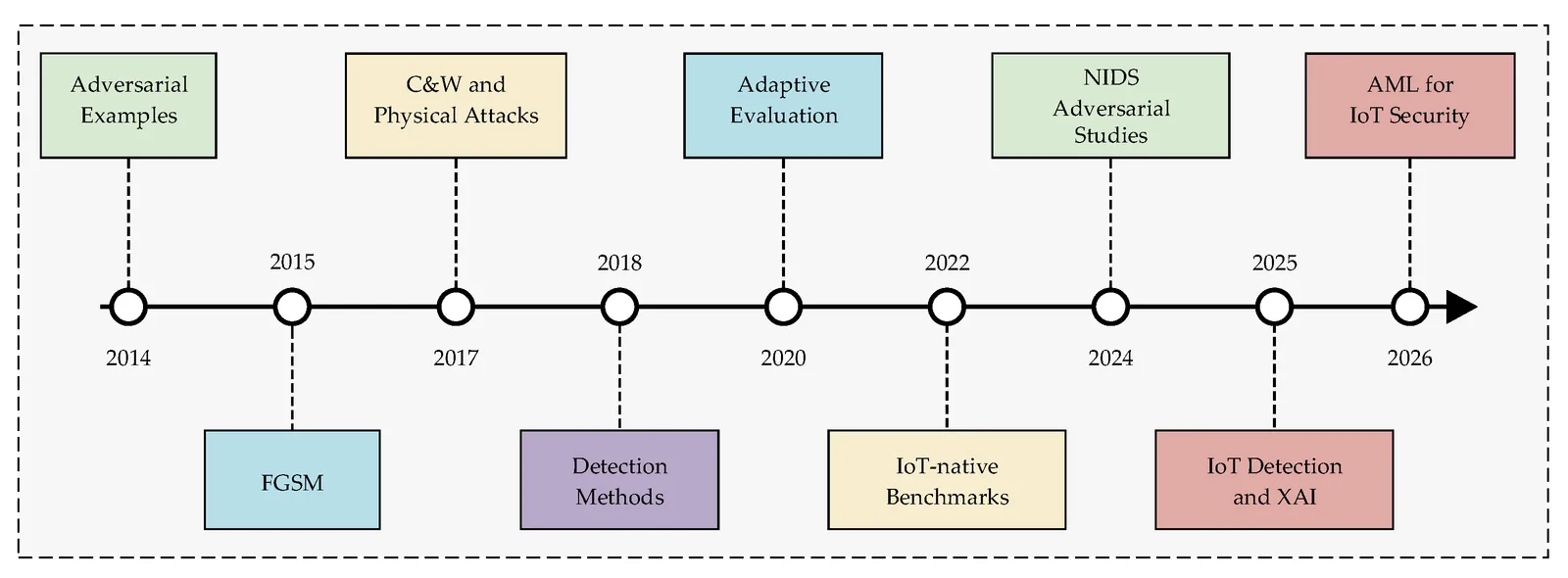
Milestone timeline of the reviewed literature.

The organization of this review is primarily based on the logical relationship between problems and evidence, while historical evolution is used only to motivate the research background. Figure 1 summarizes the main development trajectory of adversarial example detection from 2014 to 2026, including the discovery of adversarial vulnerability, the development of optimization-based and physical attacks, the introduction of different detection evidence, adaptive evaluation of detectors, and the recent focus on IoT sensing systems and network intrusion detection systems. The figure is not intended to exhaustively list all related work; rather, it illustrates how the research focus has gradually shifted from revealing model vulnerability to evaluating whether detectors remain trustworthy in realistic scenarios characterized by resource limitations, environmental dynamics, and adaptive adversaries.

The remainder of this paper is organized as follows. Section 2 defines adversary capabilities, perturbation feasibility, attack patterns, and anomaly boundaries, and introduces the seven IoT deployment constraints. Section 3 describes the literature search, screening, and analytical coding methods. Section 4 systematically reviews existing detection methods according to evidence sources and discusses the independent role of formal certification and its complementarity with empirical detection. Section 5 analyzes the relationships among threat models, evaluation metrics, detection thresholds, and adaptive attack protocols. Section 6 discusses the deployment applicability of different detection methods at the sensing layer, edge gateway layer, cloud service layer, and collaborative monitoring layer. Section 7 summarizes key research gaps and outlines future research directions and concludes the paper.

## 2. Foundations of Adversarial Detection in Sensor-Driven AI Systems

Before analyzing adversarial example detection methods, it is necessary to clarify their system environment, attack boundaries, and evaluation premises. Unlike conventional benchmark tasks such as static image classification, sensor-driven AI systems typically operate in IoT scenarios that are resource-constrained, environmentally open, data-non-stationary, and communication-intensive. Their inputs are affected by hardware capabilities, sensor calibration, environmental noise, packet loss, preprocessing pipelines, privacy constraints, and incomplete labels, and they may also be adversarially manipulated in the physical environment, wireless channel, terminal device, data stream, or feature-processing stage. Consequently, the core task of adversarial example detection is not merely to distinguish clean samples from perturbed samples, but to determine whether a detector can identify malicious input manipulation under realistic deployment constraints and distinguish it from natural drift, out-of-distribution inputs, sensor faults, and normal environmental variation.

### 2.1. Sensor-Network Intelligence and the Expansion of the Attack Surface

**Figure 2:**
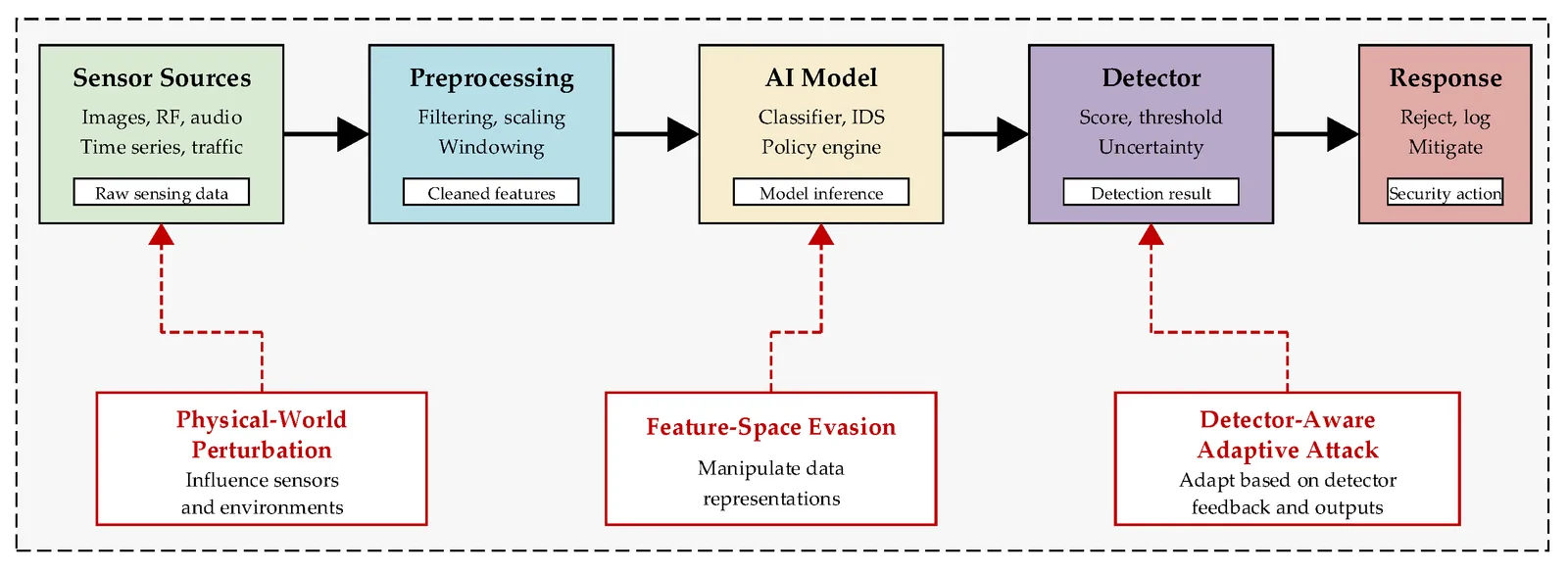
Threat model and detector deployment locations in sensor-network AI pipelines.

Traditional IoT security research has mainly focused on identity authentication, access control, encrypted communication, secure boot, firmware updates, and intrusion detection. These mechanisms primarily protect device identities, communication links, and system boundaries. Once deep learning models are embedded into sensing, preprocessing, feature extraction, inference, and feedback-control pipelines, however, the system attack surface further extends to model inputs, internal representations, decision boundaries, and detection mechanisms themselves. An attacker does not necessarily need to break authentication mechanisms or control communication protocols; by altering input data in ways allowed by physical, digital, or protocol constraints, the attacker may induce erroneous predictions, false alarms, or incorrect control decisions. Figure 2 illustrates typical attack entry points and detector deployment locations in sensor-network intelligence pipelines. Camera systems may be affected by adversarial patches, illumination changes, or scene occlusions; RF classifiers may be affected by waveform injection, channel perturbations, or hardware offsets; and network intrusion detection systems may be affected by protocol-valid but semantically malicious traffic evasion. Thus, the evidence available to adversarial detectors, the computational cost they can tolerate, and the responses they can execute are all closely tied to deployment location [23,24,25,26].

In industrial IoT and cyber-physical systems, the above attack surface is more security-sensitive. Such systems commonly have long device lifecycles, difficult protocol upgrades, strict real-time requirements, and strong production-continuity demands. Excessive rejection of normal inputs may cause production interruption, device downtime, or increased manual intervention costs, whereas unidentified malicious inputs may compromise system integrity, operational reliability, or even physical safety. Therefore, computational overhead, response latency, energy consumption, device heterogeneity, privacy protection, environmental drift, and security-response risks should not be treated as secondary implementation issues; they should be regarded as core conditions for evaluating the deployability of adversarial detection methods. Table 1 shows seven typical constraints that need to be considered simultaneously for adversarial detection in IoT scenarios. These constraints together determine whether a detector can be transferred from an experimental setup to a real system.

These constraints are mutually coupled rather than independent. Moving computation from a sensor node to a gateway can alleviate endpoint computation and energy pressure, but it may increase communication latency and privacy exposure. Raising a detection threshold can reduce the false-positive rate but may increase false negatives; lowering the threshold can improve sensitivity but may cause alert flooding, heavier operator workload, and degraded service continuity. Device-specific calibration can improve detector stability under heterogeneous deployment, but it also increases maintenance costs when the environment changes continuously. For this reason, no context-independent optimum exists for IoT adversarial detection, and effectiveness must be judged jointly in terms of deployment layer, resource budget, attacker capability, and response cost.

Another important feature of connected sensing systems is that the machine learning model is usually not the only security boundary. Real IoT systems typically include device authentication, secure boot, firmware signing, encrypted transmission, access control, intrusion detection, and human monitoring. Adversarial example detection should complement these mechanisms rather than replace all security controls. When secure boot and firmware signing reduce the probability of device compromise, runtime detectors may focus on abnormal inputs or behaviors; when network segmentation limits lateral movement, adversarial detection may prioritize high-value data streams and critical control links. From a defense-in-depth perspective, even detectors with limited standalone capability may have practical value within a multilayer security architecture [1,3,4,23,27].

The attack surface also spans the full data-management lifecycle. Training data may be collected from distributed sensors, processed through semi-automated annotation, synthetic augmentation, and periodic model updates. Data collection, labeling, cleaning, training, deployment, and updating can all introduce data poisoning, label tampering, distribution shift, or privacy leakage. Although adversarial example detection usually focuses on test-time inputs, the boundary between training-time and test-time security is not always clear in continuously learning or periodically updated IoT systems. If training data are poisoned or model updates alter the feature representation space, detectors calibrated on historical clean data may fail rapidly. Adversarial detection should therefore be considered together with data governance, model updating, and lifecycle security management [1,3,4,26,28,29].

### 2.2. Adversarial Examples and Threat Models

Adversarial examples are inputs intentionally constructed or modified by an attacker under certain feasibility constraints, with the purpose of inducing incorrect predictions, false alarms, erroneous rejection, or other security-relevant failures. In IoT scenarios, the definition of adversarial examples cannot rely solely on mathematical norm constraints; it must also consider physical realizability, protocol validity, sensor characteristics, and attacker-accessible interfaces. An analyzable threat model usually includes three elements: the attacker knowledge and access privileges, the perturbation types that can be implemented, and the system-level effects the attack seeks to achieve [30].

#### 2.2.1. Knowledge, Access Privileges, and Attack Objectives

Attacker capability is jointly determined by knowledge level, access privilege, and attack objective. A white-box attacker may know the classifier architecture, detector mechanism, preprocessing pipeline, calibration data, detection threshold, and response rules. A gray-box attacker may understand only part of the system or its approximate behavior, whereas a black-box attacker mainly relies on query feedback, transfer attacks, or surrogate models. Access privileges also differ substantially: the attacker may only be able to alter input signals in the physical environment, or may be able to inject network traffic, manipulate sensing nodes, modify data streams, or interfere with feature processing.

Attack objectives are equally diverse. Some attacks aim to induce targeted misclassification, some pursue general evasion, and others seek to generate alert flooding, delay fault discovery, degrade service quality, or trigger erroneous responses. Different objectives correspond to different success criteria. Classifier-oriented attacks focus on whether the prediction is misled; detector-oriented attacks focus on whether malicious inputs are accepted; and response-oriented attacks focus on whether business interruption, resource exhaustion, or erroneous isolation is caused. Therefore, labels such as white-box or black-box are insufficient by themselves; studies should explicitly state what information the attacker can obtain, what interfaces can be queried, what objects can be manipulated, and how attack success is determined [8,9,21,22].

#### 2.2.2. Digital, Physical, and Protocol-Constrained Perturbations

Digital perturbations act directly on the model input space and are usually bounded by norm budgets, feature ranges, or optimization constraints. They are common in image and standard classification tasks, but in IoT scenarios their feasibility also depends on preprocessing, sampling precision, and feature mapping. If a perturbation is valid only in a normalized feature space but cannot be reconstructed as a real sensing signal or executable communication behavior, its security significance is limited.

Physical perturbations must satisfy both acquisition-environment and sensing-process constraints. Adversarial patches in vision systems must remain effective under varying views, distances, illumination, and occlusions; audio attacks must consider acoustic propagation, microphone characteristics, and psychoacoustic constraints; and RF attacks are affected by channel conditions, modulation schemes, hardware differences, and noise levels [15,19,20,26,31]. Physical feasibility therefore requires not only digital-simulation effectiveness but also stable capture and attack preservation in the real sensing chain.

Protocol-constrained perturbations mainly appear in network traffic analysis and industrial communication scenarios. Such attacks must modify inputs while preserving protocol-field validity, packet ordering, feature dependencies, and malicious semantics [24,25,32,33]. Arbitrarily changing derived statistical features may produce an effective attack at the model-input level, but if the corresponding executable packet sequence cannot be generated, the attack lacks practical deployment meaning. Digital, physical, and protocol-constrained perturbations are therefore not isolated attack types; they are feasibility conditions that different attacks must satisfy during implementation.

#### 2.2.3. Data Modalities: Images, Audio, RF Signals, Time Series, and Network Traffic

Sensor data modalities determine how attack perturbations can be observed and where detection evidence remains valid. Vision detectors must handle spatial transformations, illumination changes, occlusion, and imaging-device differences. Audio detectors must follow temporal continuity, spectral structure, and psychoacoustic constraints. RF detectors must consider channel fading, hardware fingerprints, modulation schemes, and receiving conditions. Industrial time-series detectors must distinguish attack perturbations from device aging, operating-condition changes, and process drift. Network traffic detectors must preserve protocol semantics, field dependencies, and attack functionality.

These differences show that a unified classification of detection evidence is useful for comparison but cannot replace modality-specific feasibility analysis. The same detection idea may exhibit very different reliability across sensor modalities. Reconstruction-error methods may be stable in image tasks but highly sensitive to normal operating-condition changes in non-stationary industrial time series. Feature-statistics methods may show strong discriminability on centralized datasets but be affected by hardware heterogeneity and environmental drift in cross-device deployment. The effectiveness of adversarial detection must therefore be jointly defined by evidence type, data modality, and perturbation feasibility.

### 2.3. Detection Formulation and Anomaly-Boundary Definition

#### 2.3.1. Natural Drift, Out-of-Distribution Inputs, and Adversarial Examples

Natural drift, out-of-distribution inputs, and adversarial examples may all cause high suspiciousness scores, but their causes and security implications differ. Natural drift refers to non-malicious changes over time in the data-generating process, device state, or external environment, such as sensor aging, temperature variation, operating-condition adjustment, or user-behavior changes. Out-of-distribution inputs are samples outside the support covered by training data, which may arise from new devices, new environments, new classes, or abnormal operating conditions and do not necessarily imply malicious intent. Adversarial examples are inputs deliberately optimized by an attacker under explicit objectives and constraints to induce expected model or system failures.

The three phenomena can overlap observationally. Attackers may exploit natural drift to conceal perturbations or use out-of-distribution samples as attack carriers, while sensor faults may statistically resemble adversarial perturbations. Nevertheless, they can still be distinguished by intent, relation to the training distribution, optimization constraints, and expected outcomes [34,35,36,37,38,39,40,41,42,43,44,45]. Natural drift emphasizes temporal evolution and environmental change, out-of-distribution inputs emphasize insufficient training-distribution coverage, and adversarial examples emphasize deliberate optimization toward model failure.

This distinction directly affects detection evaluation and system response. Natural drift requires attention to temporal splits, device splits, and calibration stability; out-of-distribution detection requires novelty recognition and cross-distribution generalization; and adversarial detection requires attack success rate, detection-evasion rate, and residual system risk to be assessed under explicit attacker capability models. A suspiciousness score alone usually cannot determine the root cause of an anomaly, and detection results should therefore be interpreted together with response strategies. When drift, out-of-distribution inputs, and adversarial tampering are difficult to distinguish observationally, systems should prioritize secondary sampling, gateway review, multi-source cross-validation, or human escalation rather than immediately executing high-cost isolation.

#### 2.3.2. Suspiciousness Scores, Decisions, and System Utility

A base model maps inputs to predictions or anomaly scores, while a detector generates suspiciousness scores from raw inputs, transformed inputs, hidden-layer activations, gradients, output distributions, reconstruction residuals, temporal context, or system metadata. A thresholding strategy then converts the score into decisions such as acceptance, rejection, deferral, escalated review, or mitigation. Detection and robust defense therefore have different objectives: robust defense emphasizes correct prediction under perturbation, whereas adversarial detection emphasizes identifying whether the current input or prediction lacks trustworthiness.

Threshold selection is the key step that converts statistical evidence into application risk. False positives may cause service interruption, device isolation, maintenance triggers, or increased operator burden; false negatives may allow malicious inputs to enter downstream decision processes and cause security consequences. Deployment operating points therefore cannot be determined solely by aggregate discrimination metrics such as AUROC. AUROC is useful for measuring initial separability, but real systems also require concrete thresholds, false-positive tolerance, residual attack success after rejection, response cost, and processing latency. Without this information, high offline metrics do not sufficiently demonstrate engineering usability.

Suspiciousness scores are evidence rather than direct proof of anomaly causation. The same high-scoring sample may result from adversarial manipulation, out-of-distribution input, natural drift, sensor fault, or preprocessing abnormality. Detection mechanisms therefore need to be connected with hierarchical, traceable, and auditable response processes. Low-confidence evidence may trigger additional sampling, local retesting, or gateway-side review; high-cost actions such as isolation, degraded operation, standby-model switching, or human takeover should be used only when multilayer evidence is consistent. Such design can reduce the security and availability risks introduced by misinterpretation of a single detection score.

The detector information interface also affects method construction and fair comparison. Post hoc detectors may access only output probabilities or predicted labels; integrated detectors may access hidden representations, gradients, and calibration statistics; gateway detectors can use temporal context, cross-device relationships, and communication behavior; and lightweight detectors on sensor nodes may observe only local data within short time windows. If the accessible information is not reported, algorithmic ability may be confused with additional observation privileges, thereby overestimating the generality of certain detection schemes.

A complete adversarial detection system should therefore include at least five interrelated components: the base decision model, the evidence interface, the suspiciousness scoring mechanism, the thresholding strategy, and the response strategy. The base model determines the representation space on which the detector relies; the evidence interface determines the scope of observable information; the scoring mechanism quantifies abnormal evidence; the thresholding strategy converts statistical results into operational decisions; and the response strategy determines the real cost of false positives and false negatives. Changes in any component can alter the overall effectiveness of the detection system.

### 2.4. Existing Foundations and Unresolved Issues

Existing studies have discussed adversarial examples, model robustness, and security detection from multiple perspectives. General adversarial machine learning surveys mainly focus on attack generation, robust training, and defense mechanisms [46,47,48,49]. Out-of-distribution detection and uncertainty research emphasize novelty recognition, confidence calibration, and open-set generalization [34,35,36,37,38,39,40,41]. Network intrusion detection studies pay greater attention to traffic-feature feasibility, protocol constraints, and evasion attacks [24,50]. IoT security surveys usually place intelligent-model security within broader frameworks of device, communication, privacy, and system protection [51,52]. These directions are complementary, but their problem formulations, evaluation objects, and security assumptions are not fully consistent.

For sensor-driven AI systems, the central question of adversarial example detection is not merely whether a scoring function can separate two test sets, but whether the evidence on which the detector relies can be obtained under real deployment conditions, whether it can withstand evasion by attackers with corresponding knowledge and access privileges, and whether it can function under resource, privacy, latency, and security-response constraints. If these conditions are ignored, a detector may perform well on standard datasets yet lose reliability under physical perturbations, protocol constraints, device heterogeneity, or environmental drift.

Recent IoT and NIDS studies have begun to address protocol-constrained evasion, adversarial training, autoencoder-based detection, attribution fingerprints, and edge-side detection [25,33,50,51,52,53,54,55,56,57]. These works have pushed adversarial detection from static sample discrimination toward real-system adaptation, but they also reveal inconsistencies in experimental methodology. Some studies directly modify derived features without verifying whether the features can be inverted into executable traffic; some use real datasets but cover only narrow attack types; and some report aggregate accuracy or AUROC while omitting threshold, false-positive rate, latency, energy consumption, and response consequences.

From a system perspective, an IoT adversarial detector is part of a closed loop of sensing, communication, inference, and response rather than an isolated sample-filtering tool. Its evaluation criteria should simultaneously cover the validity of detection evidence, the reasonableness of the attack model, the satisfiability of deployment constraints, and the security of the response strategy. Only when all these elements are made explicit can detector performance claims become interpretable, comparable, and useful for engineering practice [58,59]. Table 2 compares this review with representative prior surveys using the same dimensions, including topic scope, IoT and sensor coverage, attack realism, edge-deployment constraints, and study-selection methodology.

## 3. Review Methodology

To improve transparency and reproducibility, this review adopts a structured search, screening, quality-assessment, and coding procedure for adversarial example detection studies in IoT and sensor-network scenarios. The search covers representative studies from 2014 to 2026, including adversarial example attacks and evaluation, detector design, uncertainty analysis, out-of-distribution recognition, network intrusion detection system (NIDS) evasion, IoT security, and edge deployment [8,9,10,11,12,13,14,15,16,17,18,19,20,21,22,24,25,28,29,32,33,34,35,36,37,38,39,40,41,42,43,44,45,46,47,48,50,51,52,53,54,55,56,57,60,61,62,63,64,65,66,67,68,69,70,71,72,73,74,75,76,77,78]. Table 3 summarizes the search process, screening results, eligibility criteria, and coding dimensions used in the review.

Because studies differ substantially in model architectures, data modalities, perturbation budgets, attacker capabilities, threshold-setting rules, and hardware environments, this review does not conduct a numerical meta-analysis. Instead, it adopts qualitative synthesis and design-oriented comparison to identify evidence categories, evaluation weaknesses, deployment constraints, and future research directions.

### 3.1. Literature Search Strategy and Data Sources

The search strategy combined terminology from adversarial learning, detection, IoT security, and network intrusion detection. It covered adversarial example detection, adaptive attacks, feature squeezing, Mahalanobis and LID detectors, uncertainty analysis, data reconstruction, adversarial NIDS, IoT adversarial machine learning, sensor-network security, and edge intrusion detection. Search platforms included IEEE Xplore, ACM Digital Library, ScienceDirect, SpringerLink, MDPI, arXiv, Google Scholar, and citation snowballing around key attacks, detectors, and evaluation studies [18,21,22,24,46,47,51]. The screening process followed a staged workflow consisting of database retrieval, duplicate removal, title-and-abstract screening, full-text eligibility assessment, exclusion based on scope and methodological sufficiency, and final coding of included studies. The search retrieved 328 records; after removing 50 duplicates and excluding 134 records during title-and-abstract screening, 144 full-text articles were assessed, 70 were excluded at the full-text stage, and 74 studies were retained for the coded synthesis. Full-text exclusions were mainly due to a lack of adversarial setting, absence of a detector or evaluation component, insufficient methodological detail, uninterpretable attack feasibility, or duplicate/superseded versions.

### 3.2. Eligibility, Screening, and Quality Assessment

Included papers were required to involve adversarial attacks, detection, evaluation, IoT or sensor-network security, NIDS robustness, edge deployment, federated detection, uncertainty analysis, data reconstruction, or related trustworthy AI mechanisms. Conventional anomaly-detection studies without adversarial relevance, defenses lacking detection or evaluation modules, and papers with insufficient methodological detail were excluded from the main comparison. Eligible studies were coded by domain, model type, attacker capability, evidence source, dataset, evaluation metric, adaptive-evaluation mechanism, and deployment applicability. The coding process explicitly recorded whether attacks were adapted to the detector. Results without detector-aware optimization were treated as baseline separability rather than robust security, consistent with adaptive-evaluation guidance [18,21,22].

The quality assessment did not use study quality as a purely numerical score because the reviewed studies span adversarial ML, NIDS, IoT security, uncertainty estimation, formal certification, and deployment-oriented systems research. Instead, each study was assessed according to whether it clearly reported the threat model, dataset and split strategy, base model, attack feasibility constraints, detector information access, threshold selection rule, adaptive-evaluation protocol, resource or latency overhead, and reproducibility information. Studies with incomplete reporting were retained when they were methodologically or historically relevant, but their limitations are explicitly reflected in the evidence-mapping table and synthesis. Screening and coding decisions were cross-checked by the authors against the eligibility and quality-assessment criteria. Uncertain cases were resolved through discussion and full-text rechecking before final coding.

### 3.3. Classification Coding and Analytical Synthesis

The synthesis emphasizes taxonomy rather than chronology. Studies were coded according to input transformations, latent representations, predictive uncertainty, reconstruction processes, runtime or protocol context, and cross-layer fusion. Representative methods define evidence categories [28,29,34,35,36,37,38,39,40,41,61,62,63,64,65,66,67,77,78], while Section 4 analyzes how each category behaves under IoT constraints. A second coding dimension covered base model, dataset, perturbation feasibility, attacker access, threshold-selection rule, and detector-aware optimization. These variables determine whether reported scores represent fixed-attack separability or credible adaptive-security outcomes [18,21,22,60]. The final synthesis is interpretive and security-centered. It does not rank detectors by a single accuracy value; instead, it identifies causal advantages, adaptive failure modes, resource conflicts, and evidence gaps. When latency, energy consumption, privacy protection, response speed, or governance mechanisms affect evaluation conclusions, evidence from IoT/NIDS research [23,24,25,27,32,33,50,51,52,53,54,55,56,57,73,74,75,76,79,80,81,82] and risk-assessment or governance frameworks [83,84,85,86,87] is considered.

**Figure 3:**
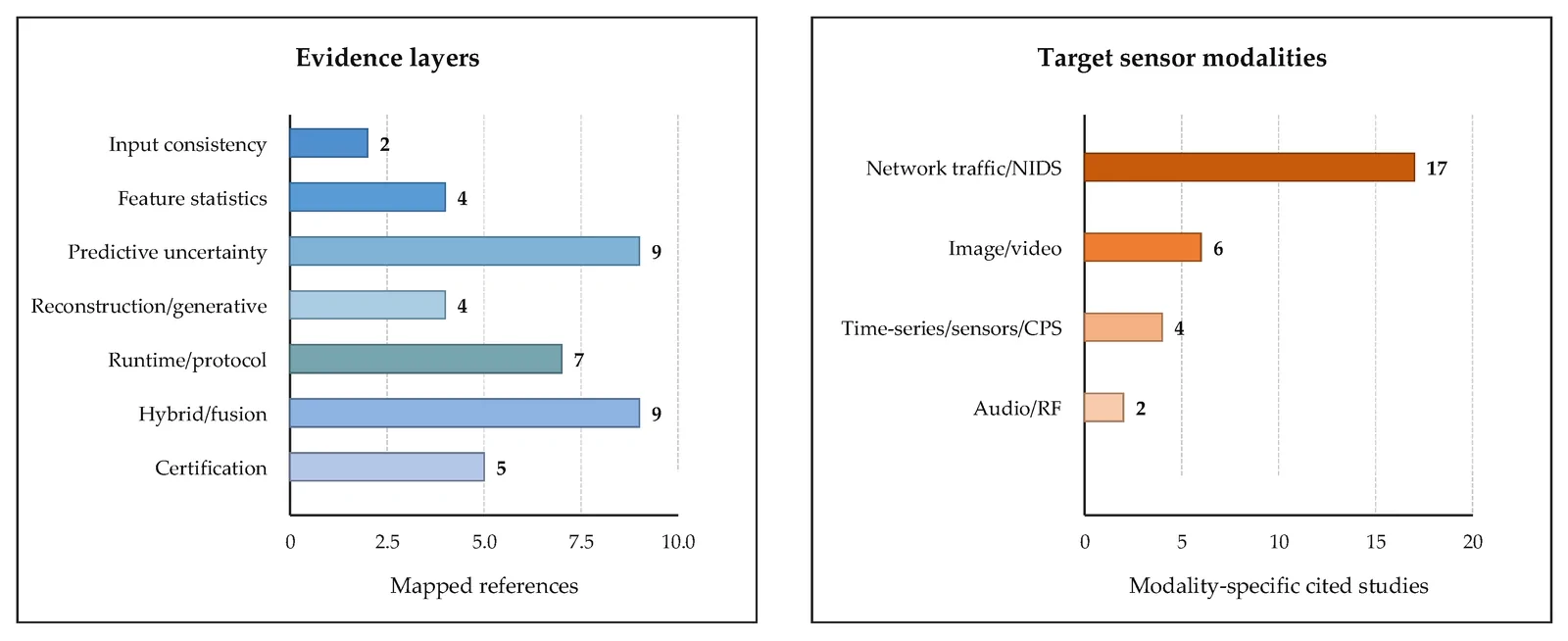
Distribution of reviewed evidence by detection evidence layer and target sensor modality.

To make the taxonomy less purely conceptual, the manuscript includes a concise representative evidence-mapping layer. Table 4 provides a concise version in the main text. This mapping connects representative studies to sensing modality, datasets, attack feasibility, deployment layer and quality concerns, thereby indicating how strongly different conclusions are supported and where evaluation flaws are concentrated. In addition, Figure 3 summarizes the coded evidence layers and target sensor modalities to make evidence distribution and modality gaps more visible.

## 4. Detection Methods and Mechanisms of Action for Adversarial Samples in the Industrial Internet of Things

**Figure 4:**
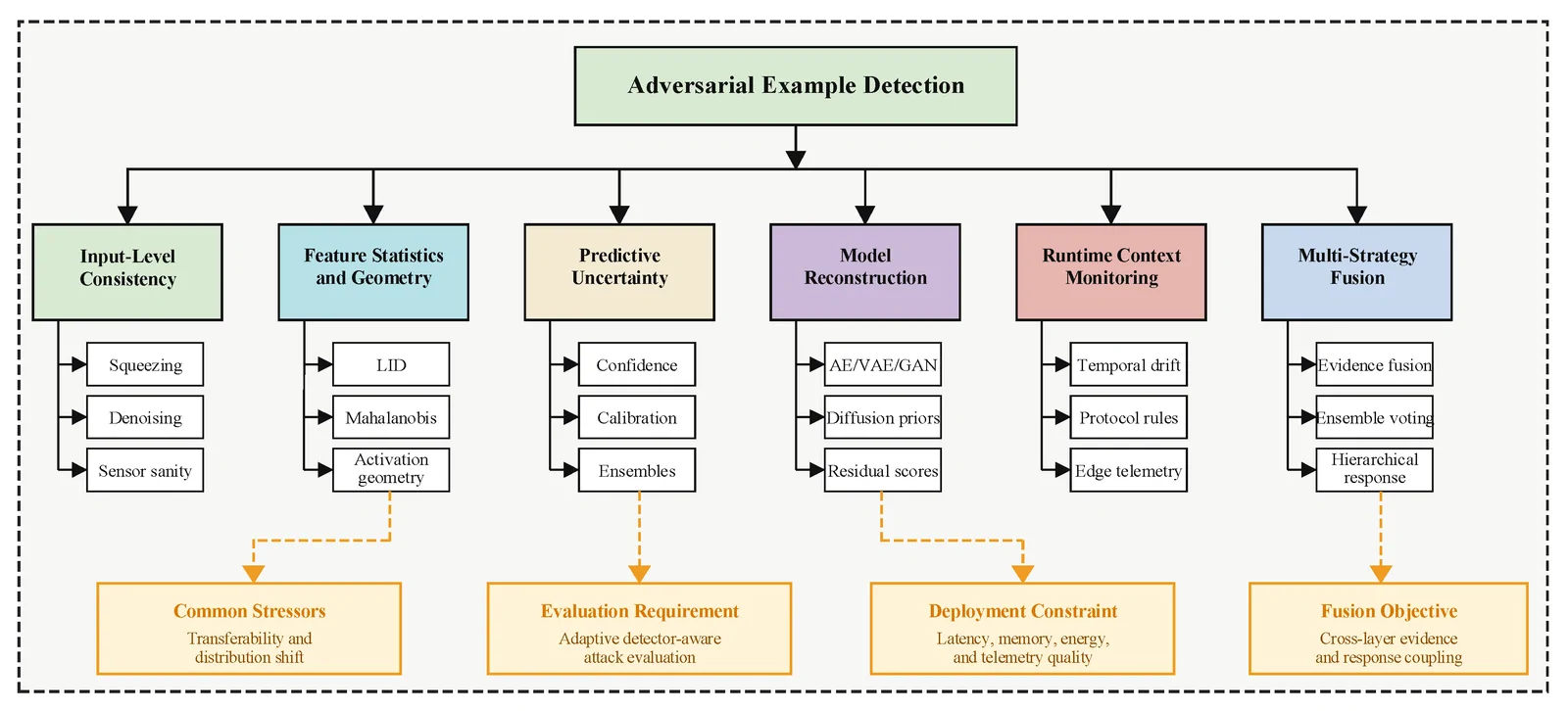
Hierarchical taxonomy of adversarial example detection methods.

According to the information sources and discrimination mechanisms used by detectors, existing adversarial example detection methods can be grouped into six categories: input-consistency detection, feature-statistics detection, predictive-uncertainty detection, model-reconstruction detection, runtime-context detection, and multi-strategy fusion detection. Figure 4 visualizes the six detection families, including five primitive evidence layers and the multi-strategy fusion layer, and Table 5 derives each family’s advantage, weakness, and deployment fit from its underlying mechanism. This classification is not centered on algorithm names but on what evidence detectors rely on, why that evidence can expose abnormal inputs, and what deployment constraints affect it in IoT and sensor-network environments.

### 4.1. Input-Consistency Detection

Input-consistency detection identifies abnormal samples by analyzing input stability under semantic-preserving transformations, physical-invariant constraints, or preprocessing operations. Its basic assumption is that normal inputs should preserve relatively stable model predictions after compression, smoothing, quantization, denoising, resampling, or mild physical transformation, whereas deliberately optimized adversarial perturbations may be disrupted by these operations and thus produce prediction changes or score anomalies. Feature squeezing compares prediction differences before and after quantization or smoothing [65], while MagNet uses manifold projection and prediction-disagreement analysis to detect samples deviating from the clean-data manifold [64].

The main advantages of this family are simple implementation and low information demand, because access to internal model features or the training process is usually unnecessary. On resource-constrained sensor nodes and edge gateways, input-consistency detection can serve as a low-cost preliminary screening mechanism. For cameras, audio sensors, RF receivers, and industrial sensors, if transformations that should preserve semantics can be clearly defined, input-level detection can filter obvious abnormal data at an early stage.

Its reliability, however, strongly depends on whether transformation design matches the data modality. Bit-depth reduction may be suitable for images but not for discrete protocol fields; smoothing can weaken high-frequency image noise but may erase short critical transients in industrial time series; range constraints can exclude obviously invalid inputs but may fail to detect subtle manipulation within the physical range. For network traffic, arbitrary feature squeezing or smoothing may also destroy protocol semantics and render detection results meaningless in engineering terms. Adaptive attacks are the main challenge: if attackers know filter parameters, transformation distributions, or randomization rules, they can incorporate these operations into the attack objective, and for randomized preprocessing they can approximate the expected gradient [18,22]. Benign device calibration, environmental drift, and sampling noise can also cause transformation inconsistency. Thus, input-consistency detection is better suited for suspicious-sample triage than for standalone high-cost security-response decisions.

### 4.2. Feature-Statistics Detection

Feature-statistics detection uses internal activations, embeddings, gradient information, local geometry, or class-conditional distances as anomaly evidence. Its core idea is that even when adversarial examples obtain high confidence at the output layer, their trajectories in hidden representation space may deviate from the statistical structure of normal samples. Hidden-layer detectors [62], local intrinsic dimensionality (LID) [28], Mahalanobis distance scoring [29], and representation-based statistical tests [66] belong to this category.

Compared with methods based only on output probabilities, feature-statistics detection observes intermediate prediction formation. This provides stronger mechanistic interpretability: such methods examine not only what the model outputs but also how the input is represented. In many deep models, abnormal inputs may be masked by high-confidence final predictions while leaving deviations in intermediate-layer geometry, class-conditional distances, or neighborhood density.

Deployment costs are also higher. Multilayer feature extraction and reference-set retrieval increase computation and memory requirements. Reference data may contain sensitive sensing information or network-traffic features, creating privacy-leakage risks. Feature distributions may drift with hardware model, firmware version, sampling condition, deployment location, and time. If detectors rely on fixed reference sets or static class statistics, thresholds and scores may fail across devices or long-term operation. In adaptive settings, feature statistics themselves can become optimization targets: attackers can jointly optimize classification error and feature-space score so that samples are misclassified while remaining close to normal representation regions. If reference sets are polluted during operation, the normal baseline may gradually shift. Feature-statistics detection therefore requires secure reference maintenance, device-aware calibration, drift monitoring, and detector-aware evaluation before it can be trusted in IoT environments.

#### 4.2.1. Neighborhood Geometry and Local Intrinsic Dimensionality

LID estimates how the number of neighboring samples around a representation point grows as distance expands. Adversarial examples may show local geometry different from normal samples, so LID can serve as a statistical signal for identifying abnormal representation paths [28]. This method is suitable for capturing abnormal changes on complex local manifolds, but it depends on sufficient and stable reference samples. Its main deployment limitations are nearest-neighbor search and reference-data storage. High-dimensional feature retrieval can create substantial computation and memory costs for resource-constrained devices, and long-term storage of raw reference features may cause privacy leakage. Device updates, environmental drift, and operating-condition changes can alter local neighborhoods and undermine existing LID scores. Practical applications should therefore specify reference-set sources, update mechanisms, memory budgets, and sensitivity to runtime changes.

#### 4.2.2. Class-Conditional Statistics and Mahalanobis Scoring

Mahalanobis scoring builds class-conditional feature distributions and measures the distance between an input representation and the nearest class statistical model [34]. Compared with LID, it usually does not require storing full neighborhood structures and compresses reference data into statistics such as class means and covariance matrices, reducing deployment cost. Multilayer Mahalanobis aggregation can also capture abnormal inputs at different representation levels. Its limitation is sensitivity to distributional assumptions and stationarity. If class features are not approximately Gaussian, or if device, environmental, or temporal factors shift class distributions, Mahalanobis scores may generate significant errors. A large class distance does not directly prove malicious intent; it may also correspond to natural drift, out-of-distribution input, or a new operating condition. Mahalanobis scoring is therefore better suited as risk-escalation evidence than as the sole basis for attack attribution. LID and Mahalanobis scoring represent two typical orientations in feature-statistics detection: LID emphasizes local neighborhood geometry and offers finer anomaly evidence at higher computational and storage cost, while Mahalanobis methods are easier to deploy but rely on stronger distributional assumptions. Neither is naturally resistant to adaptive evasion, so statistical assumptions, resource demands, and attack-evaluation conditions must be disclosed.

### 4.3. Predictive-Uncertainty Detection

Predictive-uncertainty detection extracts evidence from model outputs, including confidence, entropy, classification-boundary distance, ensemble disagreement, Bayesian uncertainty, calibration residuals, and consistency scores [34,35,36,37,38,39,40,41,44]. Its basic idea is that inputs far from reliable prediction regions should exhibit higher uncertainty, and when uncertainty exceeds a threshold the system may reject, defer, or escalate the decision. Its main advantage is low information cost: many uncertainty metrics can be computed directly from output probabilities without hidden-layer access or large reference sets. This makes predictive-uncertainty detection suitable as a lightweight trigger for resource-constrained devices, edge gateways, or low-latency scenarios. For safety-critical systems, its value lies in selective prediction and risk stratification: when the model is insufficiently confident, the system need not force a decision but may enter a more conservative process.

Prediction uncertainty, however, is not equivalent to security. Modern neural networks may remain overconfident on adversarial and out-of-distribution inputs [34,35,40,41]. Attackers can include confidence, entropy, or ensemble disagreement in the optimization objective, maintaining misclassification while reducing suspiciousness. Calibration also drifts across devices, sampling environments, and time, causing fixed thresholds to fail in long-term operation. Predictive-uncertainty detection should therefore specify calibration-data sources, threshold-setting procedures, and stability under environmental change. In high-risk IoT systems, low uncertainty should not be interpreted as proof of input safety; it only indicates that the current output distribution does not show obvious abnormality under the present model and calibration conditions. A more reliable strategy is to combine uncertainty evidence with input consistency, feature statistics, or runtime context so that a single output score is less vulnerable to shaping and evasion.

### 4.4. Model-Reconstruction Detection

Model-reconstruction detection identifies abnormal samples by evaluating whether inputs conform to learned clean-data priors. Autoencoders, variational autoencoders, generative adversarial networks, and related generative scoring methods use reconstruction error, latent-space deviation, or prediction differences between original and reconstructed inputs as detection signals [53,64,77,78]. The underlying assumption is that normal samples can be reconstructed well by the clean-data manifold, whereas adversarial examples containing abnormal perturbations produce larger residuals or prediction inconsistency. Such methods can exploit structured data priors and are especially useful when attack labels are scarce or all attack types cannot be enumerated. For images, audio, industrial time series, and some sensing signals, reconstruction models can provide anomaly evidence in unsupervised or semi-supervised settings, and they retain some openness to unknown attacks compared with detectors trained entirely on attack samples.

The limitations are also clear. Generative inference and repeated reconstruction are computationally expensive and difficult to deploy directly on low-power sensor nodes. Benign novelty, device updates, environmental variation, and operating-condition switching can also produce large reconstruction errors, making attacks hard to distinguish from normal evolution. For network traffic and protocol data, reconstruction models must preserve protocol semantics and field dependencies; otherwise, they may merely learn statistics of derived feature tables rather than the structure of executable communication behavior. Adaptive attackers can jointly optimize classifier error and reconstruction consistency so that adversarial samples both induce wrong predictions and maintain low reconstruction residuals [18,21,22]. They may also exploit blind spots in generative models to construct malicious inputs that appear to lie on the clean manifold. Reliable evaluation should therefore include reconstruction-aware attacks, benign novelty errors, inference latency, resource overhead, and residual-uncertainty handling.

### 4.5. Runtime-Context Detection

Runtime-context detection uses system-level evidence unavailable to isolated sample classifiers, including temporal consistency, state-estimation residuals, protocol invariants, cross-sensor consistency, device identity, reputation information, and graph relationships. Its core logic is contextual plausibility: an input may appear normal at the single-sample level, but if it conflicts with device history, neighboring sensors, physical-process laws, or protocol constraints, it may still carry high security risk. This family is particularly important in IoT, industrial control, and NIDS scenarios [23,24,25,32,33,50,53]. Runtime context can identify attacks that single-point models miss: a sensor reading may lie within a legal range but contradict neighboring sensors or historical states; a traffic segment may satisfy basic protocol syntax but display anomalous windows, field dependencies, or behavior sequences; and a device prediction may be confident while long-term behavior has drifted from baseline. Such evidence extends detection from whether a sample is abnormal to whether behavior is coherent in a system context.

The deployment prerequisites are reliable telemetry, historical baselines, and domain rules. Long time windows increase latency; cross-device aggregation introduces privacy and communication overhead; heterogeneous devices and protocols complicate baseline modeling; and continuous baseline updates increase maintenance costs. If attackers gradually pollute historical baselines, forge telemetry, or choose attack timing outside detection windows, runtime-context detection can also fail. Therefore, studies must specify window length, protocol validity, telemetry integrity, baseline-update rules, and detection latency. Compared with static feature-space attacks, contextual evasion in IoT depends more on temporal structure, device relationships, and protocol semantics. Without modeling these elements, detectors may only verify statistical anomalies in offline datasets rather than real attack paths.

### 4.6. Multi-Strategy Fusion Detection

Multi-strategy fusion detection integrates evidence from input consistency, feature statistics, predictive uncertainty, model reconstruction, and runtime context to improve robustness. The basic motivation is that a single evidence source usually has specific blind spots, whereas multi-source evidence requires attackers to satisfy multiple evasion conditions. Security gains come from evidence complementarity rather than from simply increasing the number of detectors. In IoT systems, fusion is usually hierarchical. Sensor nodes may conduct lightweight input screening or output-uncertainty evaluation; edge gateways may compute feature statistics, temporal consistency, and cross-device relationships; and cloud or security centers may perform high-cost reconstruction analysis, long-term trend modeling, and global correlation. This structure can partially balance endpoint resource constraints and complex security analysis.

Fusion does not automatically guarantee adaptive robustness. If multiple detectors depend on the same fragile features, they may fail under the same attack. If attackers know the fusion rule, they can construct joint objectives that suppress multiple suspiciousness scores. If some endpoint devices are compromised, attackers may forge, hide, or tamper with telemetry evidence. Fusion rules also introduce new threshold surfaces and calibration problems, which can become unstable under environmental drift or load variation. Reliable fusion requires explicit evidence-management rules: scores should be standardized on deployment data, missing evidence should have fallback mechanisms, and fusion rules should distinguish complementary from redundant evidence. Requiring multiple evidence sources to trigger jointly can reduce false positives but increase response latency; allowing a single high-severity signal to trigger response can accelerate mitigation but increase single-point error risk. Fusion detection must therefore evaluate component failure, calibration drift, node compromise, score forgery, and system overload; otherwise, multilayer architectures may merely relocate uncertainty rather than reduce security risk [18,21,22,88,89,90,91,92,93].

### 4.7. Formal Certification as an Independent Security-Assurance Dimension

Formal certification should not be treated as a seventh category of detection evidence. The six method families above focus on what anomaly cues detectors can observe, whereas formal certification focuses on what provable guarantees can be provided under specified perturbation sets and model assumptions. Randomized smoothing, convex relaxation, and reachability verification can in principle prove that, within a specified perturbation range, an input will not be transformed into an undetected successful adversarial example [68,69,70,71,72]. Such guarantees may apply to input-based, feature-based, prediction-based, or fusion-based detection systems and interact with robust training, model compression, and calibration methods in shaping detection boundaries [17,94,95,96,97].

In IoT scenarios, formal certification is more suitable as a selective or offline-assisted mechanism. Because sensor nodes are constrained by computation, energy, and latency, full online verification is often too costly. More feasible approaches are to establish local certificates for high-risk operating regions, safety-critical modes, physically interpretable perturbations, or protocol-constrained perturbations, and to compute them during design, pre-deployment testing, or cloud security analysis. This can provide stronger security evidence for critical decision boundaries without substantially increasing online inference burden. The main risk of formal certification is not ordinary detection evasion but mismatch between certification specifications and real attack conditions. If a certificate covers only norm-bounded perturbations but not channel variation, physical capture error, discrete protocol dependency, device drift, or preprocessing change, its mathematical guarantee cannot be directly translated into operational security. Model updating, pruning, quantization, retraining, or sensor replacement may also invalidate previous certificates. Certification claims must therefore specify perturbation sets, applicable modalities, model and preprocessing assumptions, uncovered attacks, computational cost, and revalidation triggers. Outside the certified region, detectors still require empirical adaptive evaluation; the value of certification is to define provable secure operating boundaries, not to replace runtime detection or system-level response.

## 5. Evaluation Protocols and Boundaries of Security Claims

Evaluation is the key stage for determining whether detector security claims hold. Many detection methods perform well under fixed attacks or limited datasets, but their performance may originate from artifacts of specific attack generators, threshold leakage from the test set, infeasible feature perturbations, or unreported deployment differences. These problems are more pronounced in IoT and sensor-network scenarios because detectors must address not only model-input perturbations but also device heterogeneity, communication protocols, physical environments, resource budgets, and response costs.

**Figure 5:**
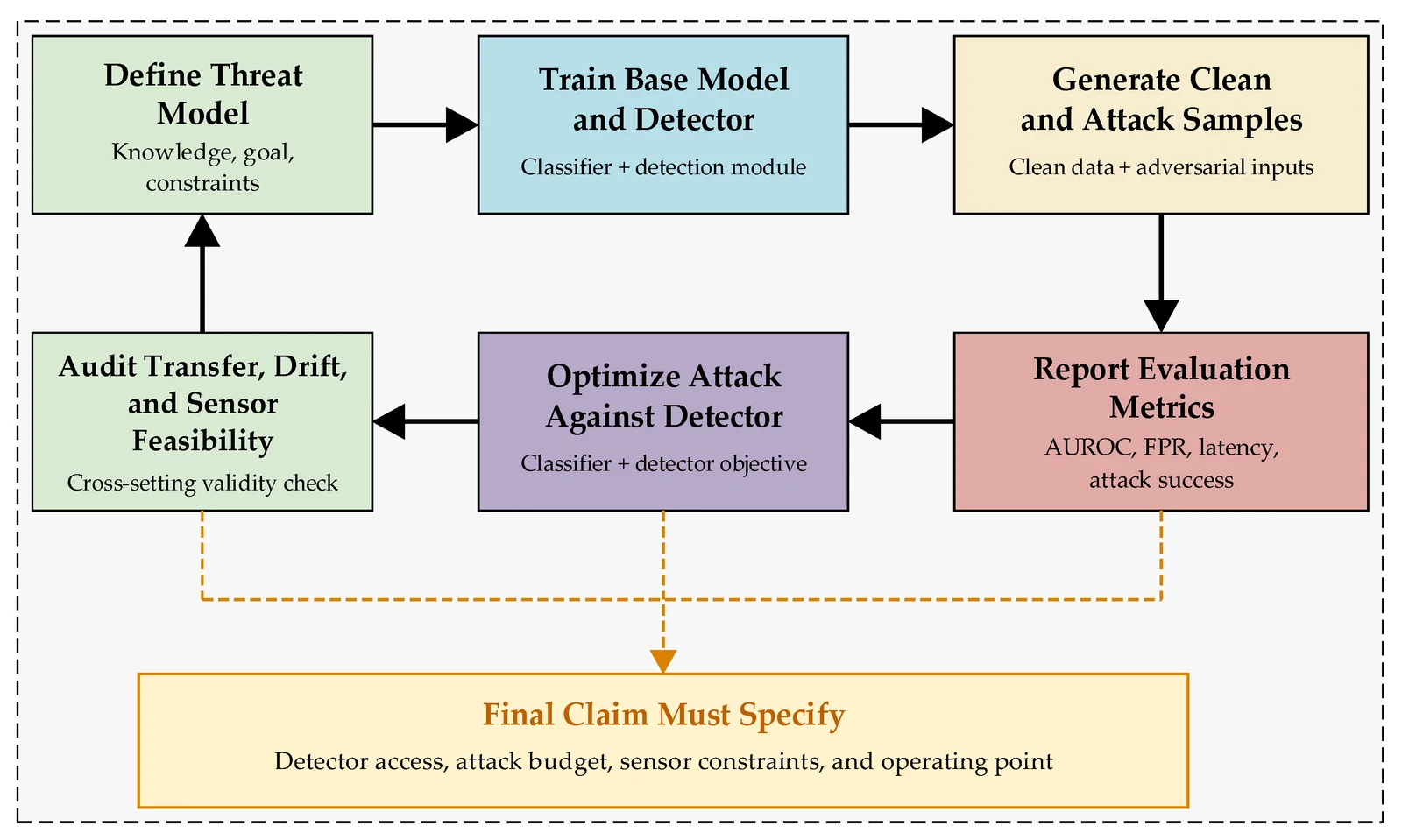
Detector-aware evaluation loop.

Adversarial detection evaluation should therefore not report only detection accuracy or AUROC; it should evaluate the complete classifier-detector system. Reliable evaluation should first specify attacker capability, perturbation feasibility, detector information interface, threshold strategy, and response mechanism. The attacker should then optimize against the joint classifier-detector decision rule. Finally, studies should report detection discriminability, residual attack success after rejection, resource overhead, transfer performance, and operational risk [18,21,22,60]. Figure 5 presents a closed-loop evaluation process for detector-aware attacks.

Comparisons among detectors must be interpreted together with threat models and deployment budgets. A detector that provides limited but stable protection under strong adaptive attacks may be more security-relevant than one with high AUROC under fixed attacks. Likewise, a fast, low-latency, low-energy screening mechanism deployed at the sensing layer may be more practical than a complex detector with higher offline accuracy but excessive cost. Reasonable evaluation criteria should include attacker capability, performance metrics, threshold setting, resource cost, and security response rather than reducing detector performance to a single statistical score.

A common validity threat is overlap between the detector and the final test-attack distribution. If a detector is tuned on the same attack generator used for testing, it may learn artifacts of that attack rather than general adversarial evidence. Robust evaluation should separate calibration attacks from test attacks; include unseen attack objectives, transfer attacks, and benign device changes; and check whether the detector conflates natural drift, out-of-distribution inputs, and adversarial examples. A method that labels all distribution shifts as adversarial attacks may achieve high benchmark metrics while lacking operational specificity.

### 5.1. Benchmark Datasets, Attack Suites, and Sensor Data Domains

Standard datasets such as MNIST, CIFAR, and ImageNet remain useful for analyzing basic detector behavior, but IoT and NIDS deployment requires data resources closer to real scenarios, such as UNSW-NB15, CICIDS2017, Bot-IoT, ToN-IoT, and Edge-IIoTset [27,79,80,81,82]. These datasets differ substantially in collection environment, feature construction, device identifiers, temporal structure, class distribution, and attack realism. Performance under random splits is therefore insufficient to prove practical deployability; more meaningful evaluation should include cross-device, cross-time, and cross-dataset splits to test stability under device heterogeneity and environmental drift.

Attack suites must satisfy feasibility requirements of specific data modalities. Image tasks can use FGSM, PGD, C&W, DeepFool, AutoAttack, universal perturbations, and physical transformations [11,13,14,15,16,17,19,20,60]. Network traffic and tabular attacks must preserve discrete fields, protocol dependencies, packet order, traffic-statistical relationships, and malicious semantics [24,25,32,33,50]. Physical sensing attacks must remain effective under real acquisition chains, environmental variation, and sensor noise. If an attack exists only in an unrealizable feature-vector space, it proves only that the optimizer can alter model inputs, not that a corresponding security risk exists in a real IoT system.

Data splitting also strongly affects conclusions. Random record splits may place near-duplicate flows, device behaviors, or time windows in both training and test sets, thereby overestimating classifier and detector performance. Splits by time, device, or deployment scenario better reflect generalization pressure in real systems. When attack samples are scarce or classes are highly imbalanced, precision-recall analysis is often more informative than accuracy alone. Dataset reports should include collection background, preprocessing, feature definitions, time-window settings, and potential leakage risks so that performance claims are interpretable and reproducible [27,79,80,81,82]. For tabular network traffic and protocol data, feasible attack generation remains a central challenge. Independent continuous-space feature optimization may violate logical relationships among packet counts, byte counts, flags, duration, and transmission rates. A reasonable attack process should include protocol validators, raw traffic replay, or feature inversion, and adaptive attacks should be subject to the same feasibility rules. Otherwise, protocol-aware detectors may merely reject impossible samples rather than identify executable attacks.

### 5.2. Metrics, Thresholds, and Operating Points

Adversarial detection evaluation should distinguish three metric categories: detection discriminability, system-security effect, and operational cost. AUROC, AUPR, true positive rate, false-positive rate, and equal error rate describe how well suspiciousness scores separate samples; attack success after rejection, robust accuracy under abstention, and residual risk after response measure whether detection actually reduces adversarial impact; latency, memory, energy, communication overhead, calibration cost, and response delay indicate whether detectors satisfy IoT deployment constraints. These categories are not interchangeable: high discriminability does not necessarily imply low residual attack risk or engineering usability.

Thresholds convert detection scores into system decisions. Different thresholds lead to different false positives, false negatives, resource consumption, and response costs. Thresholds should not be tuned post hoc on the final test set; they should be determined using clean validation data, known attack samples, or predefined risk models. Selecting thresholds by maximizing test-set F1 causes information leakage, while reporting only low-false-positive operating results may conceal adaptive fragility. Table 6 summarizes recommended metrics and reporting norms.

Metric selection should match attacker capability. For black-box or non-adaptive attacks, AUROC and AUPR can serve as baseline separability metrics, but they do not directly prove security because the attacker has not optimized against the detector and rejection rule. Such results are better interpreted as separability under fixed attacks rather than adaptive robustness [18,21,22,60]. For white-box or detector-aware attacks, evaluation must report whether the attacker can simultaneously achieve misclassification and detection evasion in the presence of the detector. The joint optimization objective, detector access, perturbation feasibility, optimization algorithm or query budget, threshold, and response rule must be specified. Samples that induce classifier error but are rejected should be counted as defended cases; samples that bypass the detector and cause wrong decisions should be counted as successful attacks. Physical and protocol-constrained attacks further require end-to-end executability and response-latency validation.

Gray-box and partially observable attacks should be treated as a continuum between black-box and white-box capability. Studies can vary attacker knowledge of preprocessing, hidden-layer structure, threshold feedback, time windows, or collaborative scores to produce capability-performance curves. These curves reveal whether security depends on hidden implementation details or degrades smoothly as attacker capability increases. For query-limited attacks, detection success should also be reported as a function of query count and the cost required to obtain detector feedback. Reproducibility is also essential: preprocessing order, attack parameters, calibration splits, threshold rules, random seeds, and hardware conditions can all significantly affect detection performance. IoT evaluations should record feature extraction from raw packets to flows, aggregation windows, sensor filtering rules, feature truncation, feasibility validators, model versions, and hardware configurations. Without these details, claims about adaptive attacks or deployment cost cannot be reproduced.

### 5.3. Adaptive-Attack Evaluation

Adaptive evaluation tests whether detection evidence remains effective when the attacker knows the detector and optimizes against it. For differentiable detectors, classification error and detection evasion can be incorporated into a joint loss; for randomized or non-differentiable components, expectation over transformation, surrogate models, score estimation, or query optimization can approximate bypass strategies. Research on gradient masking has shown that fixed attacks may create a false sense of security [18], and detector-aware evaluation further shows that attacks must target the complete classifier-detector system rather than only the classifier [21,22].

IoT security evaluation must also specify attacker location relative to sensors, gateways, models, detectors, and response channels. Full white-box access can serve as worst-case stress testing, while query access, physical contact, wireless-channel control, compromised nodes, and traffic injection may better match specific deployment scenarios. Evaluation should define attacker capability and perturbation feasibility, generate candidate attacks, jointly optimize against classifier and detector, and report the residual attack success rate, detection-evasion rate, runtime overhead, and transferability.

Failure under fully adaptive conditions does not mean a detector has no value in all practical settings; it more accurately defines the boundary of the security claim. A trustworthy detection study should state both effectiveness under restricted attacker models and failure modes under stronger attackers. The methodological error to avoid is generalizing detection success under non-adaptive attacks into a broad security guarantee. Adaptive-attack reports should include objectives, attack steps or query strategies, perturbation-feasibility validation, detector and threshold settings, randomness handling, and joint success criteria. Where necessary, transfer attacks, unseen attack types, and benign distribution drift should also be reported. Only when attack generation, detection rules, and response conditions are transparent can security conclusions be interpreted.

### 5.4. Comparative Results and Design Trade-Offs

Different detection mechanisms suit different operational scenarios, and no single evidence source dominates all IoT environments. Input-consistency detection is inexpensive and suitable for rapid screening but vulnerable to transformation-aware attacks. Feature-statistics detection uses internal representation information but depends on stable feature spaces and reference sets. Predictive-uncertainty detection supports low-cost abstention and risk escalation but may suffer from overconfidence and confidence shaping. Reconstruction detection exploits clean-data priors but may confuse benign novelty with attacks. Runtime-context detection is closer to IoT system characteristics but depends on reliable telemetry and domain rules. Formal certification provides local provable guarantees but is limited by perturbation specifications and system assumptions. Table 7 summarizes representative mechanisms, assumptions, evaluation considerations, and deployment implications; the comparison emphasizes method fit rather than a single performance ranking.

Threshold setting, performance metrics, resource overhead, and response mechanisms jointly constitute the operational decision space of a detection system. Lowering the threshold can improve the true positive rate but increases false positives, computational load, communication overhead, and manual-processing burden. Raising the threshold helps maintain service continuity and energy efficiency but may increase missed attacks. A reasonable operating point should therefore balance security risk, resource budget, and business availability rather than optimize a single detection metric.

Different IoT scenarios have different risk preferences. In industrial control systems, erroneous blocking may interrupt production, whereas missed attacks may damage equipment or cause safety incidents. High-suspiciousness samples are therefore better used to trigger gateway confirmation, redundant measurement, or safe-state switching rather than immediate irreversible shutdown. In vehicle sensing, latency constraints are stricter, and local lightweight detection should prioritize sensor redundancy, progressive degradation, or emergency protection, while complex analysis can run asynchronously. In consumer IoT, energy, privacy, and alert burden are more prominent, making event-triggered local detection and selective cloud review more feasible.

The same AUROC can correspond to different operational decisions in different systems. High-security systems may tolerate higher false positives, while systems requiring extreme service continuity may find even low false-positive rates unacceptable. Papers should therefore report operating ranges, including the attack success rate, latency, energy, communication overhead, and response volume under different false-positive budgets. Threshold selection should be prospective rather than post-test tuning: acceptable false-positive workload and response cost are set first, candidate thresholds are selected on validation data, residual attack success is evaluated under adaptive attacks, and sensitivity analysis examines stability under varying attack prevalence, response cost, and environmental drift. If abstention or escalation is supported, rejected clean samples must be included in accuracy and availability statistics; if escalation triggers a second detector, additional latency and review error rate must also be reported.

## 6. Deployment Mechanisms in IoT and Sensor Networks

Adversarial detectors become operational security capabilities only when integrated into real sensing, communication, inference, and response pipelines. A detection score alone does not constitute a complete protection mechanism; the detector must specify deployment location, observable evidence, computational resources, communication path, threshold strategy, and response semantics. IoT and sensor networks usually include a sensing layer, edge gateway layer, cloud or security-center layer, federated collaboration layer, and response layer. Different layers have different resource budgets and information access privileges, and are therefore suitable for different detection tasks.

**Figure 6:**
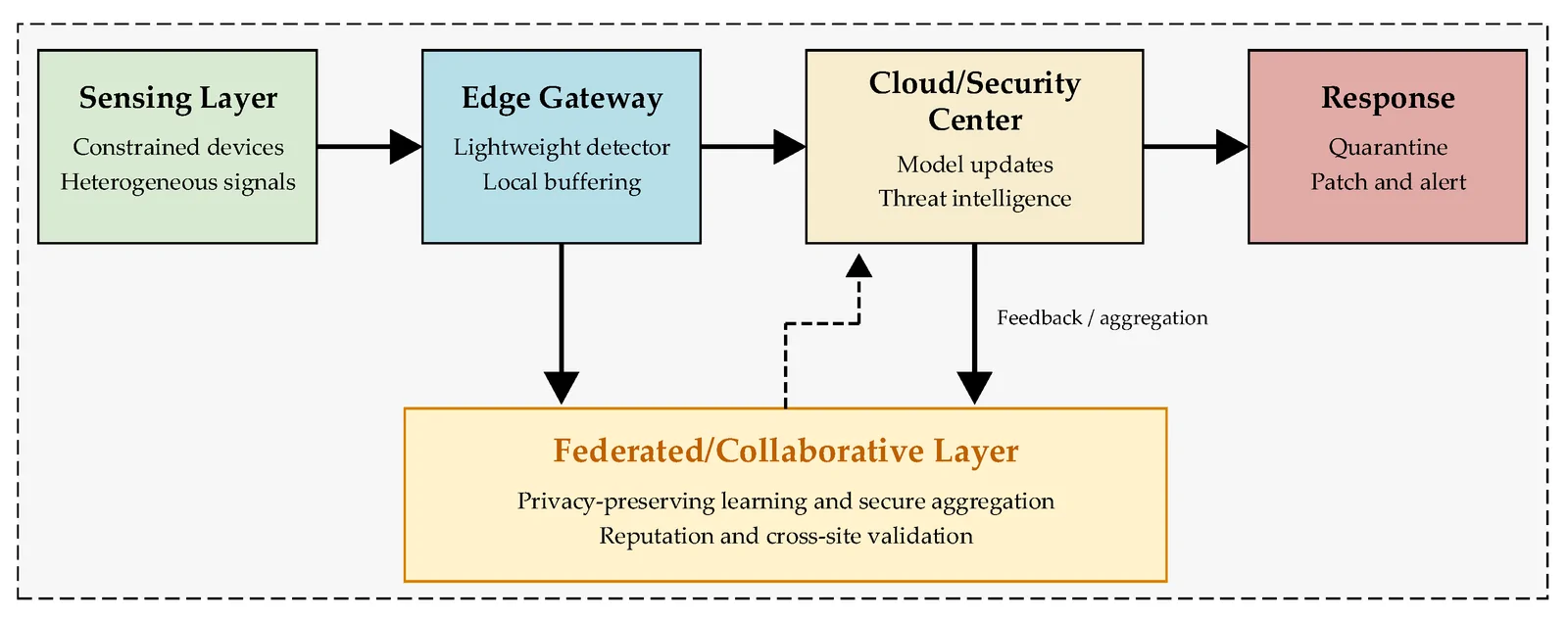
Deployment-oriented architecture for adversarial detection in IoT and sensor networks.

Detector design and evaluation are tied to deployment locality, communication cost, and response semantics. Figure 6 presents a hierarchical deployment architecture for IoT and sensor networks. The sensing layer is close to raw inputs and is suitable for lightweight input validation, simple consistency checks, and low-cost uncertainty triggers. Edge gateways offer stronger computation and local context and can support feature statistics, temporal consistency, protocol rules, and cross-device relationship analysis. Cloud or security centers are suitable for high-cost reconstruction, global correlation, long-term trend modeling, and formal-assisted verification. The federated collaboration layer aggregates cross-node summaries, distribution-drift signals, and risk scores without centralizing raw data. The response layer executes acceptance, rejection, review, isolation, degraded operation, standby-model switching, or human audit according to detection evidence.

This architecture does not imply that more complex detection systems are necessarily more secure. Sensor-node deployment offers low latency and low communication cost but is constrained by computation, memory, and energy. Gateway deployment integrates local context but introduces communication latency and privacy exposure. Cloud analysis has strong computation and global visibility but cannot always satisfy strict real-time requirements. Federated collaboration mitigates data centralization but faces node trust, model-update poisoning, and aggregation-robustness challenges. Deployment design should therefore choose evidence combinations according to application risk, resource budget, and response consequence rather than simply maximizing detector complexity.

Hierarchical deployment also introduces security costs through feedback. Suspicious samples, detection scores, and response outcomes can calibrate thresholds, update threat intelligence, and optimize models, but may also be subject to poisoning, telemetry forgery, feedback flooding, or baseline contamination. To reduce these risks, systems need provenance tracking, rate limiting, integrity verification, controlled updates, and isolation of abnormal feedback. Detector outputs should not be written unconditionally into long-term baselines, especially before attack events are confirmed. IoT adversarial detection deployment should answer four questions: at which layer evidence is generated, how evidence is transmitted and aggregated, how scores are converted into responses, and how the system prevents feedback mechanisms from being exploited. Only when these questions are addressed can the detector become an engineering-relevant security control rather than an offline scoring module.

### 6.1. Edge Computing and Resource Budgets

Edge computing can reduce detection latency and limit the privacy exposure associated with transmitting raw sensory data to the cloud; therefore, it has considerable practical value in IoT-oriented adversarial detection. However, edge-side devices are typically constrained by computational capacity, memory availability, energy budgets, and real-time requirements, making it difficult for them to continuously execute large-scale models, complex reference-set retrieval, generative reconstruction, or repeated stochastic inference. Detection tasks should therefore be allocated according to system hierarchy: sensor nodes are better suited for lightweight input checks, simple consistency verification, and low-cost uncertainty triggers; edge gateways are more appropriate for feature statistics, temporal-context analysis, and protocol-rule monitoring; and cloud platforms or security centers are better suited for high-cost reconstruction, cross-node fusion, long-term trend modeling, and offline verification.

Resource budgets are not static. During system congestion, coordinated attacks, bursty anomalies, or emergency response periods, the volume of alerts may increase rapidly, leading to queue accumulation, higher latency, and computational resource exhaustion. Under such conditions, detection procedures that are otherwise acceptable may no longer satisfy real-time operational requirements. Event-driven scoring, early-exit mechanisms, queue-aware escalation policies, and overload thresholds can help maintain service continuity, but they also change false-positive rates, false-negative rates, and response latency. Therefore, deployment evaluation should not report only average per-sample inference time, but should also include throughput under stress, worst-case latency, energy variation, and escalation capacity.

Model updates during long-term operation can also affect detector reliability. Model compression, retraining, firmware updates, modifications to preprocessing pipelines, or changes in feature definitions may alter the input distribution, latent representations, and calibration thresholds on which a detector depends. If the detector cannot be revalidated as the base model and operating environment evolve, its performance may gradually degrade after deployment. Practical systems therefore require version management, score-drift monitoring, threshold recalibration, and secure rollback mechanisms. Detection algorithms that lack such update and recalibration mechanisms may perform well in offline experiments, but they are unlikely to adequately address the long-term deployment risks caused by device heterogeneity and environmental non-stationarity.

### 6.2. Distributed, Federated, and Collaborative Detection

IoT systems are often composed of large numbers of distributed devices and sites. Different nodes may operate in different geographic locations, network conditions, and threat environments, making it difficult for a single site to observe the complete attack landscape. Distributed or federated detection allows sites to exchange model updates, compact threat signatures, uncertainty statistics, or risk scores without centralizing raw data, thereby improving the detection of rare attacks, cross-site attacks, and early-stage anomalous patterns. Federated learning provides a basic mechanism for decentralized training [88,89,90], while secure aggregation can reduce the risk of directly exposing local update information [91].

Nevertheless, collaborative mechanisms also introduce additional attack surfaces. Participating nodes may upload poisoned updates, forge threat summaries, conceal anomalous scores, or influence the global model through backdoor attacks [92,93]. Communication links may also be affected by latency, packet loss, replay attacks, or man-in-the-middle attacks. Thus, federated and collaborative detection should not be assumed to be inherently more secure. Their effectiveness depends on participant authentication, update-integrity verification, abnormal-contribution filtering, Byzantine-robust aggregation, and audit mechanisms.

#### Local Independent Detection and Federated Collaborative Detection

Local independent detection minimizes communication overhead and keeps raw data within local sites. This mode is particularly suitable for scenarios with unstable connectivity, strict privacy requirements, high site heterogeneity, or weak collaborative trust boundaries. Its advantages include short response paths, limited dependence on external nodes, and simpler data governance. Its limitation is restricted visibility: a single site may lack sufficient examples of rare attacks and may fail to benefit from early warnings observed by other sites.

Federated collaborative detection can improve cross-site threat visibility and enhance rare-pattern recognition through shared statistics or model updates. This mode is appropriate when participants are exposed to similar threat environments, can establish reliable authentication, and can support secure aggregation and auditing. Its potential costs include increased communication load, update poisoning, backdoor injection, privacy leakage, and evasion of collaborative decision rules. A reasonable design principle is therefore to preserve local autonomous decision-making by default and introduce federated or collaborative mechanisms only when the security benefits of cross-site cooperation outweigh the additional trust risks, communication costs, and privacy exposure.

The adoption of collaborative detection can follow a clear decision process. First, it should be determined whether the target attack requires cross-site evidence for identification; if local temporal context, protocol constraints, and device behavior are already sufficient, the marginal value of collaboration may be limited. Second, the trustworthiness of participating nodes should be assessed, including whether the source and integrity of their contributions can be verified. Third, the privacy and poisoning risks associated with sharing raw samples, detection scores, feature signatures, and model updates should be compared. Finally, the robustness of collaborative rules should be evaluated under malicious-client attacks, site failures, communication interruptions, and abnormal updates. Federated mechanisms are justified only when the improvement in shared threat visibility can withstand these additional failure modes.

### 6.3. Privacy, Trust, and Secure Telemetry

The evidence used for adversarial detection may contain sensitive information. Sensor readings, network-traffic features, device identities, topological relationships, user behavior, and industrial process states may all be indirectly exposed through detection scores, logs, or model updates. Therefore, detector deployment must consider security and privacy protection simultaneously. Feature minimization, local scoring, encrypted transmission, differential privacy, secure aggregation, and role-based access control can reduce information leakage to varying degrees [83,84,85,86,87,88,89,90,91,92,93].

Logging strategies must balance forensic value and privacy protection. Retaining all suspicious samples can support attack tracing, model review, and incident investigation, but it may also create a highly sensitive data repository. Conversely, retaining no samples may weaken auditing, reproducibility, and accountability. A more practical compromise is to preserve compressed evidence summaries, hash identifiers, encrypted logs, access-audit records, and limited retention periods [98,99,100]. For high-risk incidents, richer contextual information may be retained under strict access control; for low-risk events, raw data storage should be minimized.

Secure telemetry itself must also be included in the threat model. Detection scores, status logs, and risk summaries transmitted from devices, gateways, or collaborative nodes may be forged, replayed, delayed, tampered with, or selectively suppressed. If fusion-based detection or collaborative analysis depends on such telemetry while the communication path lacks authentication and integrity protection, attackers may influence final decisions by manipulating the evidence flow. Systems should therefore establish secure channels, timestamp verification, provenance tracking, integrity checks, and anomaly-consistency verification for telemetry data. A detector cannot assume that its own evidence chain is inherently trustworthy.

### 6.4. Response Semantics in Safety-Critical Scenarios

A detector’s suspiciousness score can be translated into practical security capability only when it is associated with explicit response semantics. Possible responses include deferred processing, resampling, cross-validation through redundant sensors, backup-model switching, rate limiting, deep inspection, human review, device isolation, system degradation, and conservative control. Different application domains have substantially different risk structures and cannot rely on a uniform rejection policy. Medical monitoring, industrial control, vehicle perception, and consumer IoT differ in their tolerance for false positives, false negatives, latency, and service disruption.

Response design must be validated in conjunction with domain-specific requirements. In industrial control systems, a suspicious reading may be better handled by triggering redundant measurement and a safe operating mode rather than immediate shutdown. In network intrusion detection, an alert may initiate fine-grained packet inspection and behavioral correlation before isolation. In medical monitoring, abnormal alerts often require clinical confirmation to avoid unnecessary intervention caused by false positives. The objective of adversarial detection is not simply to increase the number of alerts, but to reduce attack-induced system risk within an acceptable operational cost.

Response-aware evaluation should focus on residual risk after intervention rather than only on detector accuracy. False positives consume resources, interrupt services, and increase human workload; false negatives may allow malicious inputs to enter the control loop; delayed responses can reduce the effectiveness of mitigation; and excessive mitigation costs may undermine system availability. Detection thresholds, escalation rules, and response actions should therefore be jointly designed according to application-specific risk profiles.

From a system perspective, adversarial detection deployment involves three interdependent layers: algorithms provide anomalous evidence, the IoT environment constrains feasible computation and communication, and response mechanisms determine how detection results affect system security. Only when detection scores, resource budgets, and response semantics are jointly specified can a detector be transformed from an offline performance indicator into an executable, auditable, and engineering-relevant security control mechanism.

## 7. Conclusions

This review has addressed the problem of deep-learning adversarial example detection in IoT and sensor networks by systematically surveying related research from 2014 to 2026 and by analyzing the relationships among detection methods, threat models, evaluation protocols, deployment constraints, and security responses. A unified analytical framework centered on detection evidence sources, adversary capability models, IoT deployment constraints, adaptive evaluation, and security response mechanisms has been constructed.

Existing detection techniques have been summarized into six categories, namely input-consistency detection, feature-statistics detection, predictive-uncertainty detection, model-reconstruction detection, runtime-context detection, and multi-strategy fusion detection, while formal certification has been discussed as an independent dimension of security assurance. This framework helps clarify what evidence different detectors rely on, which sensing data modalities and deployment layers they fit, and what failure modes may arise under adaptive attacks, environmental drift, device heterogeneity, and resource constraints.

The contribution of this review is to extend adversarial example detection from a single-algorithm performance-comparison problem into a system-security evaluation problem oriented toward real IoT operating environments, thereby providing a structured reference for method classification, performance re-examination, deployment adaptation, and risk-response design. The evidence-mapping discussion further emphasizes that detection claims should be interpreted according to evidence strength, sensing modality, attack feasibility, dataset split strategy, adaptive-evaluation completeness, deployment layer, and reported quality limitations, rather than being inferred only from conceptual method categories.

Nevertheless, this review has several limitations. First, the field of adversarial example detection for IoT and sensor-network systems is evolving rapidly, and newly published studies may change the evidence landscape after the search period covered by this review. Second, because the included studies differ substantially in sensing modalities, datasets, attack assumptions, detector architectures, and reporting practices, this review provides a qualitative synthesis rather than a quantitative meta-analysis; therefore, direct performance comparisons across methods should be interpreted cautiously. Third, the evidence mapping is intended as a concise representative synthesis of the main methodological and deployment patterns, rather than an exhaustive study-by-study database. Important deployment factors such as hardware-specific latency, energy consumption, long-term environmental drift, maintenance cost, and response-policy validation remain underreported in the primary literature, which limits the strength of conclusions that can be drawn about real-world operational effectiveness.

## Figures and Tables

**Table 1:** IoT deployment constraints and their impact on adversarial detection.

Constraint	Effect on Detector Design	Practical Recommendation
Limited compute and memory	Restricts ensembles, nearest-neighbor search, and generative inference	Use hierarchical screening and deploy heavier analysis at gateways
Low latency requirements	Long inference pipelines may be unacceptable	Report worst-case latency and use early-exit policies
Battery and energy limits	Continuous monitoring may drain devices	Prefer event-driven scoring or duty-cycled checks
Heterogeneous devices	Feature distributions differ across hardware and firmware	Use device-aware calibration and cross-device evaluation
Privacy restrictions	Raw data may not be shareable	Use local scoring, secure aggregation, and minimized telemetry
Non-stationary environments	Benign drift can raise false positives	Monitor calibration drift and support threshold updates
Safety-critical response	False positives and false negatives have asymmetric costs	Tie thresholds to risk and response semantics

**Table 2:** Comparison of this review with representative prior surveys using consistent dimensions.

Prior Review	Scope	IoT/Sensor Coverage	Physical/Protocol/Adaptive Attacks	Edge Constraints and Selection Method	How This Review Differs
Akhtar and Mian [46]	Computer-vision adversarial attacks and defenses	Limited sensor coverage	Physical vision attacks discussed; detection not central	Edge constraints and selection protocol not central	Extends evaluation from vision robustness to IoT detector deployment and response policies
Yuan et al. [47]; Chakraborty et al. [48]	General deep-learning adversarial attacks and defenses	Limited IoT modality coverage	Mainly digital attacks; adaptive detector-aware evaluation limited	No structured IoT deployment screening	Adds evidence-source taxonomy, attacker-aware detector evaluation, and deployment constraints
Alatwi and Morisset [24]	Adversarial ML in network intrusion detection	Network-traffic modality	Protocol feasibility partially considered	Systematic-review orientation; edge cost not the main axis	Extends NIDS analysis to heterogeneous sensor modalities, thresholds, and response semantics
Huma et al. [51]	Adversarial ML in IoT security	Broad IoT security coverage	Discussed as an emerging issue	Selection and evidence mapping less deployment-centered	Adds study-level coding of evidence layer, modality, attack feasibility, and deployment layer
This review	Adversarial example detection in IoT sensor networks	Image/video, audio, time series, NIDS, industrial/CPS, and multimodal IoT	Detector-aware, physical, and protocol-constrained attacks considered jointly	Structured search, coding, quality assessment, and edge-deployment constraints	Integrates detector evidence, attacker-aware evaluation, thresholds, resource budgets, and response policies

**Table 3:** Search process, screening results, eligibility criteria, and coding dimensions.

Component	Description
Search dates	The review covered the literature available up to the manuscript preparation period in June, 2026.
Databases	Web of Science, IEEE Xplore, ACM Digital Library, ScienceDirect, SpringerLink, MDPI, arXiv, and Google Scholar.
Example search strings	(“adversarial example detection” OR “adversarial sample detection” OR “detector-aware attack”) AND (IoT OR “sensor network” OR “network intrusion detection” OR NIDS OR “edge intelligence”); (“feature squeezing” OR Mahalanobis OR LID OR uncertainty OR reconstruction) AND (adversarial OR evasion) AND (sensor OR IoT OR traffic).
Screening flow	Database retrieval and citation snowballing; duplicate removal; title-and-abstract screening; full-text eligibility assessment; exclusion of out-of-scope or insufficiently specified studies; final coding of included studies.
Full-text exclusion reasons	Out of scope; no adversarial setting; no detector or evaluation component; insufficient methodological detail; attack feasibility not interpretable; duplicate or superseded version.
Quality assessment	Evaluation completeness, attack-model clarity, dataset/split transparency, threshold reporting, adaptive-attack consideration, deployment relevance, and reproducibility.
Coding dimensions	Domain, sensing modality, dataset/split strategy, base model, Attack method, feasibility constraint, detector evidence, metrics, residual attack success after rejection, target deployment layer, and quality concerns.

**Table 4:** Concise representative evidence mapping for selected adversarial-detection studies.

Study/Ref.	Modality	Data/Split	Base Model	Attack/ Feasibility	Layer	Main Concern
Feature squeezing [65]	Vision /image	MNIST/ CIFAR/ ImageNet; benchmark splits	CNN classifiers	Digital transformations; adaptive transformation-aware attacks required	Device/ gateway triage	Low cost but vulnerable to adaptive preprocessing-aware attacks
LID detector [28]	Vision/ image	Benchmark image datasets	DNN classifiers	Digital adversarial attacks; reference- neighborhood dependence	Gateway/ cloud	Requires stable reference sets and efficient neighbor search
Mahalanobis scoring [29]	Vision /OOD/ adversarial	Image benchmarks; validation calibration	DNN feature extractors	Digital attacks; feature distribution assumptions	Gateway/ cloud	Can confuse distribution shift with adversarial manipulation
MagNet/ reconstruction [64]	Vision/ image	Image benchmarks	Autoencoder plus classifier	Digital attacks; reconstruction-aware adaptive attacks required	Gateway/ cloud	Benign novelty may produce large residuals
Protocol/ NIDS adversarial studies [24,25,32,33]	Network traffic/IoT NIDS	UNSW-NB15, CICIDS, Bot-IoT, ToN-IoT, Edge-IIoTset; split strategy varies	ML/DL NIDS models	Feature-space and protocol-constrained evasion; feasibility often underreported	Gateway/ security center	Attack executability, feature inversion, and raw-traffic validity often incomplete
Recent IoT adversarial detection [33,51,52,53,54,55,56,57,75,76]	IoT/NIDS/ edge security	IoT traffic datasets; often dataset-specific	Autoencoders, GNNs, robust NIDS, attribution models	Adaptive and practical constraints vary by study	Edge/ cloud/ federated	Evidence strength depends on evaluation completeness, deployment validation, and independent replication

**Table 5:** Layered taxonomy of adversarial example detection methods.

Family	Detection Signal	Strengths	Typical Weaknesses	IoT Relevance	Refs.
Input-level consistency	Prediction changes under filtering, squeezing, denoising, randomized transformations, sensor sanity checks	Simple, model-agnostic, low-cost	Can be bypassed by adaptive attacks; sensitive to benign sensor shift	Useful for device or gateway triage	[64,65]
Feature-space statistics	Hidden activations, distances, local geometry, class-conditional scores	Uses richer evidence than output confidence	Needs internal access and calibration data; may drift	Strong candidate for edge gateways	[28,29,66,67]
Uncertainty and calibration	Confidence, entropy, ensembles, Bayesian uncertainty, conformal scores	Cheap and easy to integrate with abstention	Neural models may remain overconfident; thresholds can drift	Useful for selective escalation	[34,35,36,37,38,39,40,41,44]
Reconstruction /generative	AE, VAE, GAN, diffusion residuals, prediction disagreement	Supports unsupervised settings and structured signals	Novelty can mimic attacks; generative inference may be costly	Useful for cloud or high-capacity gateways	[53,64,77,78]
Runtime/ protocol-aware	Temporal consistency, device behavior, protocol invariants, graph relations	Exploits IoT-specific context	Requires domain engineering and reliable telemetry	Highly relevant for NIDS and industrial IoT	[23,24,25,32,33,50,53]
Hybrid/ ensemble	Combined evidence from several layers	Can reduce single-signal failure	Harder to calibrate and evaluate adaptively	Fits hierarchical edge-cloud architectures	[18,21,22,88,89,90,91,92,93]

**Table 6:** Recommended evaluation metrics and reporting practice.

Evaluation Aspect	Recommended Reporting	Reason
Detection discrimination	AUROC, AUPR, FPR at fixed TPR, EER	Curves alone do not define deployment operating points
System security	Attack success after rejection, robust accuracy with abstention	Measures whether detection actually reduces adversarial impact
Threshold selection	Validation source, target false-positive budget, sensitivity analysis	Prevents test-set threshold leakage
Adaptive robustness	Detector-aware objective, attacker access, query budget, transfer tests	Non-adaptive attacks often overestimate security
Deployment cost	Latency, memory, energy, communication overhead	IoT detectors must fit resource budgets
Distribution shift	Temporal split, device split, cross-dataset testing	Benign drift can mimic adversarial evidence
Response policy	Reject, escalate, quarantine, fallback, log, human review	Detector value depends on downstream action

**Table 7:** Representative detection approaches, design implications, and supporting references.

Approach	Core Assumption	Evaluation Caution	Deployment Implication	Refs.
Feature squeezing	Perturbations are less stable under simple transformations	Evaluate against transformation-aware attacks	Low-cost first-stage screen	[65]
LID	Adversarial samples occupy atypical local geometry	Needs stable reference set and efficient neighbor search	Better at gateways than tiny devices	[28]
Mahalanobis	Clean class features follow separable distributions	Distribution shift can look adversarial	Needs recalibration in changing IoT environments	[29]
Bayesian/ensemble uncertainty	Suspicious samples induce abnormal uncertainty/ disagreement	Overconfidence remains possible under attack	Useful for abstention and escalation	[37,38,41]
Autoencoder reconstruction	Adversarial/anomalous samples reconstruct poorly	Benign novelty can increase residuals	Works when clean signal manifold is stable	[53,64,77,78]
Protocol-aware NIDS	Valid attacks must obey protocol constraints	Feature attacks must be feasibility-constrained	Important for realistic IoT network defense	[24,25,32,33]
Certification	Defined perturbation sets can be bounded formally	Certificates may not match physical/ protocol constraints	Promising for safety-critical subsets	[68,69,70,71,72]

## Data Availability

No new experimental dataset was created in this review. The literature sources discussed in the paper are available from the cited publications and public repositories where applicable.
